# Barriers and needs in paediatric palliative home care in Germany: a qualitative interview study with professional experts

**DOI:** 10.1186/1472-684X-9-10

**Published:** 2010-06-02

**Authors:** Saskia Jünger, Tania Pastrana, Martina Pestinger, Martina Kern, Boris Zernikow, Lukas Radbruch

**Affiliations:** 1 Department of Palliative Medicine, RWTH Aachen University Hospital, Pauwelsstraße 30, 52057 Aachen, Germany; 2 Department of Palliative Medicine, Malteser Hospital Bonn/Rhein-Sieg, Von Hompesch Str. 1, 53123 Bonn, Germany; 3 Vodafone Foundation Institute and Chair of Children's Pain Therapy and Paediatric Palliative Care, Clinic for Children and Adolescents Datteln, Dr.-Friedrich-Steiner-Straße 5, 45711 Datteln, Germany

## Abstract

**Background:**

In North-Rhine Westphalia (Germany) a pilot project for an extensive service provision of palliative care for children and adolescents has been implemented. Accompanying research was undertaken with the aim to assess the status quo of service delivery at the outset of the project and to evaluate the effects of the pilot project. As part of the research, barriers and needs with respect to paediatric palliative home care in the target region were explored.

**Methods:**

Semi-structured interviews with 24 experts in the field of paediatrics, palliative and hospice care have been conducted and were analysed by qualitative content analysis.

**Results:**

Four main categories emerged from the interviews: (1) specific challenges and demands in palliative care for children and adolescents, (2) lack of clear legal and financial regulations, (3) gaps in the existing care delivery, and (4) access to services. Generally the interviews reflected the observation that the whole field is currently expanding and that certain deficits are temporary barriers that will be resolvable in the medium-term perspective.

**Conclusions:**

Predominant barriers were seen in the lack of clear legal and financial regulations which take into account the specific challenges of palliative care in children and adolescents, as well as in a shortcoming of specialist services for a local based care provision throughout the federal country.

## Background

In Germany, since April 2007 the social legislation anchors in law the individual right to specialist palliative home care. Corresponding contract negotiations are being undertaken in all federal states of Germany for adult palliative care as well as for paediatric palliative care. For several reasons, the implementation of paediatric palliative care bears specific challenges [1,2]. In different countries, approaches and models of care delivery have been developed in order to improve an extensive care for children with life-limiting diseases [3-6]. The literature consistently concludes that a well-coordinated multiprofessional care network is most suitable for the provision of outpatient palliative care [7,8]. In North Rhine-Westphalia, a federal state in the North-West of Germany, a pilot project was implemented in 2007 for the development and implementation of paediatric palliative home care services [9]. For this purpose, two specialist centres for paediatric palliative care have been set up in the two regions North Rhine and Westphalia-Lippe, each with a multiprofessional team of nurses, physicians, and psychosocial staff. Their task is the coordination of existing services and the establishment of regional care networks, as well as consultation and provision of specialist care for children and adolescents with a life-limiting condition. Evaluation of the project included the description of the status quo of service delivery at the outset of the pilot project, as well as the evaluation of the activities of the specialist centres throughout the project period. Here the assessment of challenges and needs from the expert perspective is described. Methods and results of other assessment areas are reported elsewhere [10-12].

North Rhine-Westphalia has an area of 34.088,31 km^2 ^with 17.996.621 inhabitants (population density: 528 inhabitants/km^2^), among which 3.662.949 children. It is the German federal country with the highest population density and a high proportion of children and adolescents. However, the population is not equally distributed across the country. Some parts, especially the region of Westphalia-Lippe, are characterised by more rural and less populated areas. The region of North Rhine and the industrial area around the rivers Rhine and Ruhr have a more urban structure; the latter is one of the most populated regions of Europe.

In the German health care system primary care for children, especially for babies and infants, is usually provided by paediatric specialists working in general practice, even if it may also be provided by a general practitioner. Paediatric inpatient services are provided by specialised hospital departments. Palliative and hospice care is an emerging subspecialty in Germany. Health care costs are covered by the mandatory health insurance as part of the social security system, though psychosocial and volunteer services often are not reimbursed and depend on fundraising and donations.

The objective of the present study was the appraisal of the infrastructure of palliative home care delivery for children and adolescents in North Rhine-Westphalia from the experts' perspective. Particular aims were to identify the main deficits and gaps with respect to the availability of services, the access to services and the coordination of care delivery, as well as to seek potential improvements for the enhancement of service delivery.

## Methods

Interviews with experts from the fields of paediatrics, palliative care and hospice care were chosen to get access to specialist knowledge and experience with respect to the subject in question [13]. In this study, an expert is defined as a professional with a pioneer status and with a long-standing expertise in the field of paediatric palliative or hospice care. Within this project evaluation, also parents whose child had died from a life-limiting condition have been interviewed by means of a focus group and qualitative interviews in order to assess the quality of care delivery from their perspective. The results are reported elsewhere [10,11].

A semi-structured interview guide was designed in a way that allowed for an open approach of barriers and needs. During the interviews the *Critical Incident Technique *(CIT) was employed in order to allow the experts to reproduce salient effective and ineffective aspects of the existing care delivery [14,15]. The interview partners were asked for care situations which were an example of well-organised care delivery, as well as for examples which in contrast illustrated shortcomings and gaps within the existing care delivery. In addition, subjects that emerged in the course of the interviews were enquired or specified against the background of key issues for the provision of paediatric palliative (home) care identified by previous literature search [6,8,16-20]. Moreover, demographical data and information regarding the professional role, function, and background of the experts were documented. At the end of the interview, the participants were asked to indicate one or more further experts who could contribute to the data collection.

For the selection of interview partners, a pool of experts was established taking account of the heterogeneity of the different professions and organisational structures in the field of paediatric palliative care. The majority of experts have been suggested by the Advisory Board of the pilot project. An additional internet search aimed at identifying experts from areas that were not covered by this initial pool, for example the head of a self-help organisation for bereaved parents. From this pool, the final sample has been chosen step-by-step by theoretical sampling. In theoretical sampling, the selection of experts depends on preliminary results and saturation of data [21]. The experts chosen in this study were supposed to supply different information due to their specific position within the field of palliative care for children and adolescents. Saturation was reached when no new or contrasting results emerged from the interviews.

Ethical approval was conceded in June 2007 by the Ethics Commission of the RWTH Aachen University Hospital. Experts were contacted by a cover letter and an information flyer which informed them about the background and the aims of the research, the expectancies with regard to the interview, and about their specific significance for this study. Two experts denied participation due to time constraints and two refused to participate for unknown reasons. In total, 24 experts from the fields of paediatrics as well as hospice and palliative care were interviewed (Table 1); the experts had been active in their professional field, on average, for 14 years (range 7 - 28 years). The interviews were conducted in the time from August 2007 until July 2008 and were accomplished in the experts' work environment. At the outset of each interview, informed consent was obtained. The interviews were conducted by a trained interviewer (SJ) and lasted between 30 and 120 minutes with an average length of 90 minutes. All interviews were digitally recorded and transcribed verbatim.

**Table 1:** Sample of experts in paediatric palliative care and related work fields (n = 24)

TYPE OF SERVICE Interview partner	N
**Funeral undertaker**	**1**
Head funeral undertaker (m)	1

**Outpatient children's hospice service^1^**	**4**
Coordinator outpatient children's hospice service (f)	3
Board member (f) of the association of outpatient children's hospice services *(Deutscher Kinderhospizverein e.V.)*	1

**Outpatient paediatric palliative care service^2^**	**2**
Head paediatric palliative care team (physician, f)	1
Head paediatric palliative care network (social worker, f)	1

**Self-help organisation**	**3**
Chairperson (m) of the association for bereaved parents (Verwaiste Eltern e.V.)	1
Chairperson (f) of an initiative for psychosocial support for migrant families of a child with a life-limiting disease	1
Head (m) coordination "help for self-help" of a self-help organisation for people suffering from cystic fibrosis	1

**Mobile paediatric nursing team**	**2**
Head mobile paediatric nursing team (f)	2

**Paediatrician in his own practice**	**5**
Paediatrician (m) in his own practice	3
Paediatrician (f) in her own practice	1
Chairperson (m) of the regional professional association of paediatricians	1

**Inpatient clinical care**	**3**
Head of paediatric inpatient oncological department (m)	2
Senior physician inpatient intensive care unit (f)	1

**Outpatient clinical care**	**1**
Head social-paediatric centre^3^	1

**Psychosocial/spiritual care**	**3**
Hospital chaplain (f)	1
Psychosocial service of inpatient oncological department (social worker, f)	1
Psychosocial service of inpatient oncological department (psychologist, f)	1

TOTAL	**24**

^1^Outpatient children's hospice service (*ambulanter Kinderhospizdienst*) is a volunteer service for psychosocial support of the families and does not provide medical or nursing care

^2^Outpatient paediatric palliative care service (*Kinderpalliativteam*) consists of physicians, nurses and psychosocial staff

^3^Social-paediatric centres (*Sozialpädiatrisches Zentrum, SPZ*) are interdisciplinary outpatient services for the support of children with (suspicion of) developmental disturbances

The coding procedure of the interviews strictly followed the methods of qualitative content analysis [22]. A combined model of inductive and deductive coding was used, whereas deductive coding was based on the key issues for paediatric palliative (home) care previously identified in the literature [13,22]. The analysis was supported by a software programme for text analysis (MAXQDA^®^). Transcripts were initially free-coded by the interviewer (SJ) according to content, and then organised into thematic units that were continually re-visited and revised. The criteria for this revision were (a) that categories were clearly distinguishable and overlap was avoided and (b) that the hierarchy of categories and sub-categories was coherent. A code system with key categories was developed by the interviewer (SJ) and revised by two independent coders experienced in qualitative text analysis (MP, TP). The following aspects were considered in this critical review: (a) the way of coding text passages, (b) the content and the appropriateness of the specific categories and (c) the clear distinction of the categories.

In the results section, quotations from the interviews are used for the illustration of categories. The quotations were linguistically smoothed without changing the content; expletives or repetitions were deleted and replaced by ellipses (...).

## Results

Four main categories emerged from the interviews which are of crucial importance for the quality of care delivery in paediatric palliative home care in North Rhine-Westphalia:

▪ Specific challenges and demands in palliative care for children and adolescents

▪ Lack of clear legal and financial regulations

▪ Gaps in the existing care delivery/need of expansion

▪ Access to services

### Specific challenges and demands of palliative care for children and adolescents

The specific challenges and demands of paediatric palliative care are given high importance in the interviews; they are often mentioned in differentiation to adults' palliative care (Table 2).

**Table 2:** Specific challenges and demands of paediatric palliative care

SUBCATEGORY	DESCRIPTION
Time demand	High time demand for paediatric palliative care (in comparison to adult palliative care) due to involvement of the whole family system and longer illness trajectories

Basic conditions of care	Small caseload in paediatric palliative care and a heterogeneous range of conditions bear practical and economical challenges for extensive specialist infrastructure

Challenges for staff	Work in paediatric palliative home care as a "tightrope walk" due to the traumatising impact of the dying of a child and the involvement of the whole family system

Qualification	Requirement of specialist qualification in the field of paediatric hospice and palliative care, as well as supervision and staff support

Locations of care	Advantages of home care ("normality" and intimacy within familiar environment) vs. advantages of inpatient care (safety, relief of medical and nursing responsibility)

The experts mention a high time demand for paediatric palliative home care given the broad focus of care by involving the whole family system. Moreover, the illness trajectories in the majority of cases are longer in children with a life-limiting disease compared to adults who are in need of palliative care. Generally, the experts deplore that the health care system at present does not meet the children's needs and priorities. The small caseload in paediatric palliative care and the heterogeneous range of conditions make it extraordinarily difficult to offer an extensive specialist infrastructure, both from an economical point of view as for practical considerations.

In addition, paediatric palliative care is associated with specific challenges for the staff. In contrast to adult palliative care, the parents as responsible caregivers are the main contact persons, especially in infants. This aspect is reinforced by the traumatising impact of the dying child on the parents. Some experts from the field of nursing home care report that paediatric palliative home care often goes along with tensions in the domestic environment. Moreover, particular demands in the communication with the family are described, including the recognition of the parents' authority, as well as an appropriate involvement of the child or adolescent.

*"In the care for families with dying children, I think you walk on a tightrope, and from time to time you slip off with one leg". (Social worker (f), Head Psychosocial Service, Oncology)*.

Therefore, the experts underline the need for a special qualification, supervision and education of staff due to the special circumstances of paediatric palliative home care.

As to the location of care, both advantages and limitations of the care and the dying in the home environment are mentioned. The main advantages are a higher level of "normality", familiarity and intimacy within the familial environment. It means a relief for the whole family system not to use up all resources in the organisational balancing act between inpatient clinic and the every-day-life at home. Moreover, age-appropriate activities - possibly together with siblings - are more an option compared to an inpatient environment. In the experts' opinion these advantages are associated with a stabilisation of the child's condition. At the same time, the care in the domestic environment may meet medical and technical limits and can represent too high a burden for the parents in terms of nursing and psychological strains. The experts report the observation that often parents turn to hospital in times of symptom crises or in the anticipation of a tragic dying process (e.g. suffocation in dyspnoea or bleeding to death in leukaemia).

*"Perhaps the parents could have taken their child home, but they were simply unsure and said 'We do not dare it.' And this must be permitted. We should not claim that children must generally die at home. Instead of creating such a maxim, I rather prefer to leave this open to the parents." (Social worker (f), Head Psychosocial Service, Oncology)*.

Some experts mention that the stay in hospital is not always experienced as a burden; in contrast, given the close connection to a familiar clinical environment and relationship with staff it can offer security and social contacts that would not be warranted at home.

Altogether, the results of the expert interviews with regard to the location of care show that it is important to offer flexible individual decisions which take into account the particular needs of the child and the family.

### Lack of clear legal and financial regulations

A predominant issue in the interviews is financing of paediatric palliative home care (Table 3).

**Table 3:** Legal and financial regulations

SUBCATEGORY	DESCRIPTION
Adequate and transparent regulations	Lack of clarity with respect to legal and financial regulations

Continuous need to struggle	Families and professionals continuously need to struggle for services and support, associated with a high additional burden and a lack of emotional and existential stability for the families

Ethical considerations	Reflection about the gap between the wish to give the best possible care to a dying child and the financial restrictions to certain care options

Funding of specific services	Shortcomings in the funding of particular services, e.g. nursing home care, medication & medical supplies, reimbursement of paediatricians, coordination & psychosocial care, supervision & education, self-help

Overall, general shortcomings in funding are described. An even more severe notion is the lack of adequate and transparent regulations throughout the different areas of care delivery. This leads to lack of clarity, exhausting individual negotiations for each single case and the perception of arbitrariness and dumping. The consequence of this lack of transparency is described as a persistent need to struggle for the child's appropriate therapies and medical supplies. In addition, an unequal treatment of patients depending on their individual health insurance is deplored as illustrated by the quotation below. In the German health care system, the reimbursement of certain therapies and medical supplies depends on the regulations of the individual health insurance company. Moreover, there are considerable inequalities between the services and reimbursement policies of private health insurance companies (approximately 20% of the German population) and public/statutory health insurance companies. In order to improve the families' existential situation, the experts in this study demand regular reimbursement from health insurances and more specifically the payment by lump compensation - and not as usual per services delivered.

"It must be a standard benefit of every health insurance company for every child. This is not self-evident, yet. And families should be entitled to choose who will care for them. For example, today it happened that we wanted to assume the care for a child and we had to negotiate with the health insurance company. And they said: one is cheaper than the other, so we grant the cheaper one. (...) Ultimately, we have to define standard rates that are equal for everyone and that are adjusted to the paediatric demands". (Physician (f), Head outpatient children's palliative care team)

Besides the general funding deficits, shortcomings with respect to specific areas are stated. These comprise shortfalls in the funding of specialist nursing home care, medication and medical supplies, as well as the reimbursement of paediatricians providing home care. The lack of regular funding for coordination and case-management is perceived as a serious deficit. These remits are only financed - if at all - in pilot projects or temporary funding, although they are regarded as pivotal for the quality of care.

### Gaps in the existing care delivery

The interview statements on the implementation of paediatric palliative home care mainly express a need of further expansion rather than gaps. The whole field is currently perceived as continuously growing; therefore, a lot of deficiencies are perceived as temporary barriers that will be resolvable in the medium-term perspective (Table 4).

**Table 4:** Gaps in the existing care delivery

SUBCATEGORY		DESCRIPTION
Sensitisation/public awareness		Requirement of transparent information and knowledge about palliative care for children and adolescents

Specialist paediatric nursing		Lack of specialist paediatric nurses and the availability of qualified nursing services

Pain therapy/qualified medical care		Deficit of physicians qualified in specialist paediatric pain therapy and paediatric palliative medicine

Psychosocial support		High importance of psychosocial support in order to meet the complexity of care, emotional needs/isolation, cope with the families' financial emergency, offer practical/organisational support and give attention to siblings

Networking		Different levels of networking: case-independent networking, coordination/case-management, clarification of roles and interfaces, professional exchange/helpers conference

Well-organised care at home		Management of the interface between inpatient and outpatient care, anticipatory care planning, safety for the parents, challenges in contact with parents

Infrastructure		Local proximity of care, (more) specialist teams, permanent key contact person, continuing home care by inpatient service in charge, continuing care for young adults, respite care facilities

#### Advocacy work and public awareness

The experts report a need for sensitisation for paediatric palliative care; suitable facilities and authorities must be employed and families should get a chance to represent in the media. The confrontation with pain, suffering and the perception of finiteness related to this field is described as a prerequisite for openness towards palliative care in children and adolescents.

*"And I don't believe that it's due to my person that only very very few physicians call for consultation. That's really absolutely isolated cases who call and say 'I need your help now'. That is very rare and (...) even in our own unit [oncology] when I approach a colleague and say 'Gosh, this patient is a palliative patient, did you already take this and that measure into account, or shouldn't we at least talk about that?' - than very often I get the reply 'No, he is still receiving this and that [curative treatment] and here we absolutely do not talk about "palliative", yet.'". (Physician (f), Head outpatient children's palliative care team)*.

#### Psychosocial support

A lack of psychosocial support is frequently mentioned. The situation of a family with a severely ill child or adolescent is typically characterised by an interaction of emotional distress, social isolation due to feelings of shame or insecurity on part of the environment, practical challenges in the organisation of care and a social/existential emergency. Therefore, it is important to consider the whole family system in order to meet the complexity of the situation.

*"The circle of friends and acquaintances mostly is reduced dramatically. Marriages get into trouble and all of a sudden every person in the environment is confronted with death. (...) And this kind of threat, this kind of uncertainty in the face of an environment that is not prepared to deal with this situation... this raises sensibilities or uncertainties." (Coordinator (f) children's voluntary mobile hospice team)*.

#### Care infrastructure

Throughout the interviews, requirements on the care infrastructure are emphasised; these include local proximity of care, as well as a permanent key contact person. With regard to local proximity of care distances of not more than 50 kilometres or half an hour driving distance are regarded as reasonable. In this context, the need for (more) specialist centres is emphasised; experts stress the importance of a specialist paediatric palliative care team in the regional proximity. Two centres for the regions North Rhine and Westphalia-Lippe are evaluated as by far too few for the provision of care in the whole federal country.

#### Continuity of care

Continuity of care is often associated with the option of further home care by the team of the specialist hospital department in charge. A permanent key contact person is associated with more efficient communication and care planning, but also with emotional aspects like mutual trust, security and tranquillity for the family system. Further advantages of the continued home care by the inpatient service in charge are that physicians and nurses are already familiar with the child's symptomatology which warrants a higher level of safety in the domestic environment. The interface between home care and possible future hospital stays can be arranged more efficiently. However, until now no legal and financial basis for this model does exist.

*"I think that the problem (...) is when children are ripped out of their care team in the midst of their dying process. And that is also what parents pretty often say, that they do not want that. They want (...) to remain in the same care team, with the same faces they know and that they have become familiarised to for years." (Head (m) inpatient paediatric oncological department)*.

#### Networking

Both the need of case-specific coordination, as well as networking across cases with multiprofessional exchange is emphasised. Resulting advantages as a clarification of roles and a smoother transition between inpatient and outpatient care are described. Helper/carer conferences are recommended for a better flow of communication and decision-making, as well as for clear agreements and demand-oriented modification of treatment regimens. Networking also is regarded as an essential prerequisite for the early involvement of palliative care. A better organisation of care in the domestic environment according to the experts includes anticipatory planning in terms of arrangements, anticipation of possible symptom exacerbations (e.g. seizures or dyspnoea) and crises in order to avoid emergencies and unwanted hospital admissions, as well as knowledge of the conditions on site with an impression of the familial environment, in order to place the care on a secure footing.

*"For example, in the care of a child with a brain tumour we have discussed: okay, now he is at home, what can we expect? He will have seizures, he will have pain. What if it happens in the weekend, at night, or whenever? How can we make sure that medication will be quickly available? (...) Who knows about the prescriptions, and where is this documented? We cannot warrant continuous care if we only arrange something selectively." (Coordinator (f) children's palliative care network)*.

This quote shows that the early organisation of an individual care network is pivotal for an anticipatory care planning: for example, in the run-up to a discharge from hospital, a sustainable network of local carers, early organisation of medical supplies, and 24/7 on call service is indispensable for effective care at home. The feeling of security on part of the parents in this context plays an important role. This includes the confidence that every medical treatment that is necessary can also be provided for their child in the domestic environment, trust in the carers' network and self-confidence in their own ability in the handling of the child.

### Access barriers

Access to services is stated as central challenge for paediatric palliative home care. The experts describe various access barriers (Table 5).

**Table 5:** Access barriers

SUBCATEGORY	DESCRIPTION
Identification of palliative care need	Prognostic insecurity, as well as scarce knowledge about the indication of palliative care in children and adolescence

Level of awareness of different services	Lack of information on hospice and palliative care services and their potential benefits

Time of access	Reflection about the right time of involvement of palliative care in the disease trajectory

Access path	Reflection about the suitable way of access given the heterogeneous conditions and respective responsibilities within the health care system

Reluctance on part of the parents	Association of palliative care with "giving up" their child; concerns about the right balance between own commitment for their child and involvement of other carers

Special access requirements	Specific conditions for the access to services, i.e. transition to adulthood, urban vs. rural areas, migration background, malignant vs. non-malignant diseases

The deficit of clear and high-quality information on existing services for families as well as for carers is described as an important barrier. Moreover, unclear and diffuse responsibilities can inhibit access because carers do not know exactly where to refer to.

Some experts raise the concern that the terminology of hospice and palliative care may be deterring or preventing access, as well as non-acceptance on part of the parents. Information flyers or an internet portal may provide comprehensible information and increase the level of awareness on part of the parents. Furthermore, a regional network or round tables are suggested by several experts to improve the reciprocal awareness of carers and services. General transparency in the experts' view could be enhanced by information events, by the media, as well as by word-of-mouth recommendation.

Finding the adequate time to inform a family about palliative care services is regarded as a challenge by the experts. Even if there is agreement that palliative care should be involved early, ideally even at the time of diagnosis, this time might just be inappropriate because of the emotional impact and the excessive demands placed on the families to process a huge amount of new information at once. The path of access is also specified; the experts agree that in most cases a proactive facilitation of the access by a familiar key person is desirable since families often do not know the different services and feel disoriented between all their new challenges and burdens.

In addition to general access barriers, specific requirements are reflected. These include more difficult access for patients in rural areas and for families with migration background, as well as for children and adolescents with non-malignant conditions. Children with some diagnoses may not necessarily have regular contact to a hospital department or physician in charge, for example in case of multiple disabilities or certain metabolic diseases. This might make their awareness of palliative care services more difficult.

## Discussion

### Specific challenges of paediatric palliative care

Even if the specificity of paediatric palliative care was not explicitly addressed by the interviewer, this issue recurrently came up throughout the interviews. Although in the newer literature paediatric palliative care is referred to as a specialised field of its own [23-26], the results of this study clearly reflect the experts' appraisal that these specificities and the respective needs are not sufficiently met by legal and financial regulations.

Due to the small number of cases paediatric palliative care infrastructures are challenged to provide an area-wide care which is at the same time cost-efficient. The results in this study confirm the conclusions in the literature that access to services in rural and remote areas is more difficult [8,27] because the infrastructure is less complete and travel times are longer.

### Locations of care

In the international literature, generally a strong plea for the home as most appropriate location of care for severely ill children and adolescents is held [18,28]. In agreement with the findings in this study, the normality of family life and relationships, as well as the prevention of isolation, are mentioned [28-30]. At the same time the results in this study substantiate the observation that care at home has its limits and in certain situations is not desirable or possible [29]. Parents are confronted with a high medical and nursing responsibility [30,31]. Some families feel more secure in the familiar environment of a hospital ward [30]. In every single case the preferred place of care - and after all, also the place of death - should be discussed openly in terms of informed consent [23]. Recent studies suggest that it is not pivotal *where *the child is being cared for, but rather that the process of decision making on the location of care is transparent and considers the medical facts, as well as the family's values, hopes and the available resources [32-34].

### Funding

The experts in this study express a prevailing need for clear legal and financial regulations. Craft and Killen in their recommendations to the English Ministry of Health come to similar conclusions; the authors advocate a legal regulation instead of short-term solutions and single donations [35]. In addition to the exhausting emotional and existential distress that a severely ill child causes for the families, the daily struggle with agencies and health insurances often amounts to a full-time job for the parents. Since the lack of coordination of services and the access requirements of certain budgets and services add to the burden, families often desire a key coordinator who takes on the negotiations with institutions and acts in their interest [28].

### Availability of specialist services

#### Nursing care

The deficit in the provision of qualified ambulant paediatric nursing stated in this study corresponds with field observations of the current developments to establish regional paediatric palliative care networks. This shortcoming is attributed to three aspects: a general lack of qualified paediatric nurses, a lack of funding for special palliative interventions that are not acknowledged within the existing regulations, and a general reluctance to employ a specialist paediatric nursing service since in the majority of cases the specialist care as such is more expensive or causes higher costs due to longer journeys.

#### Medical care

Until now there is also a clear shortcoming of pain management and medical treatment. This finding is in line with the situation in other countries. England can be regarded as gold standard in the development of palliative care; however, recent publications report that there were only four physicians with a specialist paediatric palliative care qualification in 2003 [36] and five in 2006 [26], even though more were planned. A review by Liben et al. shows that the integration of the growing knowledge in the field of paediatric palliative care into medical curricula is a great challenge [37]. Practising paediatricians often feel insufficiently prepared for end-of-life care in children and adolescents. However, the recent developments in public awareness and in the German health care policy cautiously suggest a change of trend. For example, the establishment of a chair for paediatric pain therapy and palliative care in North Rhine-Westphalia in 2008 and another chair for paediatric palliative care in Bavaria in the same year mirrors the growing appreciation of this field as a subspecialty on its own.

#### Psychosocial support

The results of the study reveal that currently the palliative care for children and adolescents does not acknowledge the complexity of the families' psychological and social emergency. Some experts clearly state that psychological support cannot be separated from a safeguarding of the families' social existence because families are only able to confront emotional issues when existential stability is warranted. Even if by now the provision of psychosocial support by specialised staff is undoubtedly recognised as an essential component of a paediatric palliative home care, the existing psychosocial services for children and adolescents with life-limiting diseases to date generally are either not financed at all or only in terms of pilot projects without a long-term budget.

#### Transition to adulthood

The gap in the transition of care of adolescents with life-limiting diseases who reach adulthood deplored by the experts in this study is in line with the statements of Craft and Killen in their report for the English Ministry of Health - either a young adult is abruptly referred to an adult service, or he remains in a paediatric service longer than appropriate [35]. A specific ACT guideline [38] provides recommendations for the collaboration between paediatric and adult services in order to facilitate the transition and support young people in their adaption to the critical life situation. However, there is a shortage of adult services that are adequately prepared to meet these young patients' needs [35] - an observation align with the findings for North Rhine-Westphalia.

#### Access barriers

The access barriers identified in this study have also been stated in previous research [28,36,39]. A lack of information on existing services, as well as reluctance on part of the families, is likely to impede access to palliative care services. Often families are exhausted by the coordination of the daily care for their child and may therefore experience it as an additional burden to be confronted with an unmanageable number of carers [28]. Moreover, the utilisation of hospice and palliative care services might be associated with abandoning their child. Unwillingness to recognize the incurable condition of their child has been identified as the second most common barrier to palliative care, next to insecure prognosis, in a recent survey [39]. An insufficient communication about the severity and the course of the child's condition can impede adequate decision-making and early organisation of care in the home environment [36].

In addition, it might be useful to develop specific care pathways for different diseases and conditions. For example, experts from the oncological field point out the demand for a legal and financial basis that would allow them to continue the care for their patients after discharge at home, which currently is not possible in Germany [40]. At the time when palliative care should begin, the families often are not able to accept new services. In this fragile phase the majority does not wish to involve new carers in their family system, but instead turns to those professionals who have accompanied them throughout the disease of their child. The advantages of continuity of care are expressed in terms of effective communication and care planning, as well as emotional security. Studies suggest that an equal recognition of informational continuity, management continuity, and relational continuity can help to foster and monitor continuity within a care network [41,42].

### Strengths and limitations of the study

The strengths of the data collection in this study are a broad spectrum of experts and an open access to the issues that emerged from the interviews. Rigour of data analysis was ensured by a strict protocol for the coding process. However, a weakness of this study is that no interrater reliability was calculated for the independent review of the code system.

In correspondence with the qualitative methodology and the explorative approach of this study, the experts have been systematically selected by means of theoretical sampling. Herein, a highest possible information density has been aimed at with respect to different professions and geographical regions in the federal country. However, the expert sample is not representative. Some aspects have been investigated less intensively than their importance within this field might have suggested. For example originally interviews with experts from several self-help organisations for specific life-limiting diseases had been planned. However, the coordination of appointments with some selected representatives turned out to be unexpectedly complicated: target dates were repeatedly postponed, delegated to other representatives of the same organisation or in one case cancelled without replacement. Along with this, these experts expressed a view that the respective disease actually is not at all related to a need for palliative care. This attitude mirrors the existing uncertainty regarding the various potentialities and indications of palliative care in different conditions and patient groups.

For organisational reasons the process of data collection lasted almost one year until the analysis of the interviews could start. This study has taken place in a time of rapid developments and in a phase of substantial upheaval in paediatric palliative care in Germany. Therefore, some statements must be considered with caution, because even within this short period changes in the care delivery can have occurred. For example, parallel to this pilot project significant developments have taken place throughout the whole federal country of North Rhine-Westphalia, initiated by the political discussions around the implementation of specialist palliative home care in the German social law and the increasing attention towards the field of paediatric palliative care. A growing awareness for the need of multiprofessional and intersectoral cooperation has led to the formation of local networks in other regions of North Rhine-Westphalia. Moreover, this pilot project itself, as well as the accompanying research, have made a contribution to public awareness and have boosted the perception of this field in stakeholders, decision makers, affected families, carers and in the general public. Therefore, some perceptions possibly describe temporary challenges rather than persistent and enduring shortcomings.

## Conclusions

The results of the study reflect the appraisal that the whole field of paediatric palliative care in Germany is currently expanding and that certain deficits are temporary barriers that will be resolvable in the medium-term perspective. Predominant barriers were seen in the lack of clear legal and financial regulations that take into account the specific challenges of palliative care in children and adolescents, as well as in a shortcoming of specialist services for a local based care provision throughout the federal country. Sensitisation and public awareness and close networking of local players were recommended for facilitation of the families' access to services. Enhancement of the care infrastructure was regarded as a prerequisite for proximity of care, continuity of care, and consequently a sustainable provision of paediatric palliative care in the domestic environment.

## Competing interests

The authors declare that they have no competing interests.

## Authors' contributions

SJ elaborated the methodology of the study including the interview guide; she managed the data assessment, carried out the expert interviews, did the qualitative content analysis and wrote the article. TP and MP made substantial contributions to the analysis and interpretation of data, as well as to the reporting of data. MK supported the study by specific knowledge in the area of palliative home care in Germany. BZ contributed to the selection of appropriate experts for the data assessment, supported the interpretation of data with his specialist expertise in the field of paediatric palliative care and provided relevant literature suggestions. LR developed the basic conception of the study; he supervised the entire study process and gave important advice at all steps of the research project. All authors read and approved the final manuscript.

## Pre-publication history

The pre-publication history for this paper can be accessed here:

http://www.biomedcentral.com/1472-684X/9/10/prepub
